# Estimating Long-Term Care Costs among Thai Elderly: A Phichit Province Case Study

**DOI:** 10.1155/2018/4180565

**Published:** 2018-01-17

**Authors:** Pattaraporn Khongboon, Sathirakorn Pongpanich

**Affiliations:** ^1^College of Public Health Sciences, Chulalongkorn University, Pathumwan, Bangkok 10330, Thailand; ^2^Prince Mahidol Award Foundation under the Royal Patronage, Faculty of Medicine, Siriraj Hospital, Bangkoknoi, Bangkok 10700, Thailand

## Abstract

**Background:**

Rural-urban inequality in long-term care (LTC) services has been increasing alongside rapid socioeconomic development. This study estimates the average spending on LTC services and identifies the factors that influence the use and cost of LTC for the elderly living in urban and rural areas of Thailand.

**Methods:**

The sample comprised 837 elderly aged 60 years drawn from rural and urban areas in Phichit Province. Costs were assessed over a 1-month period. Direct costs of caregiving and indirect costs (opportunity cost method) were analyzed. Binary logistic regression was performed to determine which factors affected LTC costs.

**Results:**

The total annual LTC spending for rural and urban residents was on average USD 7,285 and USD 7,280.6, respectively. Formal care and informal care comprise the largest share of payments. There was a significant association between rural residents and costs for informal care, day/night care, and home renovation.

**Conclusions:**

Even though total LTC expenditures do not seem to vary significantly across rural and urban areas, the fundamental differences between areas need to be recognized. Reorganizing country delivery systems and finding a balance between formal and informal care are alternative solutions.

## 1. Introduction

Compared to other Asian developing countries, Thailand is in serious need of long-term care (LTC) due to its rapid aging population [1]. Since an average Thai survives beyond 80 years, LTC should be increased to nearly tenfold from 2000 to 2050. Unlike earlier cohorts, the population of Thailand and other developing countries, who would reach 70–80 years in the next few decades, are expected to be prone to noncommunicable diseases [2, 3]. According to the World Health Organization (WHO), LTC includes activities carried out by formal caretakers such as professionals; auxiliaries like social, health, or other workers; or informal caregivers like neighbors, friends, and family [4]. LTC policies are different in different nations and are influenced by culture, history, structure, and economic performance [5, 6].

The government of Thailand completely understands the challenge that LTC poses regarding the declining availability of family aid [7, 8]. In 2011, a new community care policy was developed as a part of a project in the district of Lam Sonthi, in Lopburi Province [9]. Additionally, a funding of USD 17.4 million was created by the National Health Security Office (NHSO) for LTC facilities for the elderly [10]. In the fiscal year 2016, a trial program announced by the government spread across 1,000 subdistricts, including 100,000 severely disabled individuals [11].

Previous studies in Thailand show that the average operating cost of NGOs' nursing homes in 2007 was USD 271 per resident, while the average monthly operating costs of government institutions were USD 313–361 per resident. The average monthly expenses were USD 748 per resident, which were paid by private nursing homes [1]. In Bangkok, the average monthly expenditure of private institutions' LTC was USD 464.45 per person–USD 406 in the northern areas and USD 638 in the southern areas [12]. According to Wongsin et al. [13], the total cost per day of LTC-dependent elderly citizens in hospitals in 2012 was USD 130,624 annually and that of formal care assistants was USD 12,191 annually. The average expenditure of institutional LTC for the elderly moderate ADL dependence was USD 13–13.35 per person daily.

Considering physical and social health, rural and urban residents receive different access to health care services [14, 15]. Due to dualistic urban-rural economic structure, there are vast inequalities among both rural and urban areas in spite of the rapid economic growth [16, 17]. The progress of health care expenditures is of particular concern to a rural population whose incomes are lower than their urban counterparts [17, 18].

Studies conducted in other countries show that health care and LTC costs were related to factors like age, gender, comorbidity, admission, dependence in personal activities of daily living (ADL), living arrangement, and health status among others [19–21]. To be specific, the aforementioned demographic factors may exert a significant influence on the scope of an individual's access to care, exclusively related to residential care placement. For example, an individual's income and health insurance might help them gain easier access to health care. Similarly, there are other sources of help such as social support networks and publicly financed LTC. Individuals who do not have the adequate economic backup or health insurance support can seek help from the aforementioned formal service providers within the health care service sector [22].

One can easily decipher that functional impairment is generally regarded as the factor behind the failure to indulge in ADLs. Against this backdrop, those who are in need of health or personal care services confront the problem of ADL impairment; for instance, those who deal with household works tend to depend on the aforementioned services. On the other hand, one can expect extreme ADL impairment and related assistance from formal and informal bases within the parlance of long-term health care [23]. Besides, senior citizens with ADL impairment, but living with their relatives, are less likely to be placed in any health care facility, including nursing homes. On the other side, loner unmarried senior citizens with ADL impairment are usually taken care of by their relatives or friends [24].

Regional differences can be seen in the context of utilizing health care facilities, especially within the supply of LTC choices like patient care facilities at nursing homes [25]. Research works conducted on elderly citizens who tend to live in their own homes prove that men usually seek help from informal sources, whereas women tend to seek help from formal sources [21]. Similarly, one's marital status can determine the range of assistance within ADL impairment treatment. In this scenario, research premised upon community involvement proves that family support is an essential factor which determines the level of assistance sought by married individuals, divorcees, and widowers. Besides, married individuals tend to seek formal assistance as compared to their unmarried counterparts in general [24].

The policy care for Thailand has been formed using a costing model that takes into account projected expenditure and actuarial estimates to bring about various LTC options [13, 26]. However, no research has been undertaken on the difference in LTC expenditures between rural and urban areas. In addition, when the LTC provisions for Thailand are planned, influencing factors should be understood together with LTC service costs. With this information, policy-makers can understand the health care needs of elderly citizens and their families and will be able to establish an appropriate LTC system.

This study is not focused on the LTC policy under the National Health Security funding. Assuming that the elderly are the primary payers and that the rural elderly have higher LTC spending than those in urban areas, the average spending on LTC services is estimated and the factors influencing the use of LTC in urban and rural areas are extracted.

## 2. Materials and Methods

### 2.1. Study Area

A transversal survey was conducted with the help of in-person interviews in Phichit Province during February to June 2016. Phichit is situated in the north of Thailand. Of its population, 21.1% are aged 60 years and over, which is higher than the national average (14.9%). Based on the 2015 National Civil Registration Systems [27], the area in Phichit with the highest percentage of people aged 60 and above was Bang Mun Nak subdistrict (19.44%) followed by Muang subdistrict (18.29%). So, in this study, Bangkmulnark subdistrict represents rural areas and Muang subdistrict represents urban areas.

### 2.2. The Number of Subjects or Sample Size

The sample size of the elderly was determined by adopting the Taro Yamane formula for minimum sample size [28]. The minimum sample size was 364 in the rural area and 333 in the urban area. The nonresponse rate was 20%, and thus, the sample size in the rural and urban area increased to 437 and 400 people, respectively.

### 2.3. Recruitment of Subjects

A simple random sampling was employed by computerized random selection of the elderly names from the National Civil Registration list. The subjects were recruited from the community in subdistrict (Tambon) areas, Phichit Province. The interviewing team made contact with the community headman prior to visiting each selected subject at their home. The subjects comprised elderly people and their main caregivers, all of whom were willing to participate and gave their consent. The inclusion criteria were (1) being 60 years old or above, having the ability to talk with clarity, and being in possession of one's senses and (2) living alone or with someone who was assumed to be a caregiver and had served in that role for at least 1 month.

### 2.4. Survey Tools and Data Collection

Suwanrada et al. [12] designed the structured interview-administered survey used in this study. The survey, which had open-ended as well as closed-ended queries, included queries regarding the sociodemographic characteristics of elderly people (age, place of stay, education, salary, gender, single or married, lifestyle, and health details). In addition, the survey asked whether elderly respondents had any sort of disability, which it described as problems carrying out any of 10 normal tasks (taking a bath, eating food, maintaining personal hygiene, going to the toilet, climbing stairs, getting dressed, walking 200 meters, moving in bed, urinary incontinence, and fecal incontinence). Moreover, the survey inquired about the caretakers' characteristics (education, age, salary, number of hours devoted to caregiving, and gender).

Furthermore, the survey asked subjects for the details of items bought in the previous month, including the price of medical equipment (e.g., hearing aid, glucometer, bedpan, bubble mattress, back brace, and bathing chair), the price of everyday goods (e.g., adult diapers), the cost of medical procedures (out of pocket), lifestyle, travel, and home renovation for elderly during the year.

### 2.5. Data Quality

Skilled research associates conducted the data collection. Data management tasks were carried out to monitor the quality of the information. At the end of every interview, the examiner checked whether the data collected were complete. The data were entered twice into SPSS 18 data. To enhance their quality, data-cleaning techniques were used. Moreover, where data were not included, SPSS 18 displayed the term “system-missing.”

### 2.6. Cost Component

In this study, the cost component consisted of both direct and indirect costs:

- Direct costs are expenses that can be directly traced to a product. The direct costs that the elderly people incurred were out-of-pocket medical expenses for the previous month, which covered doctors' appointments and medications, medical devices, everyday necessities, and proper care.
- The indirect costs associated with LTC are mainly related to the loss of human capital of carers. The indirect costs of the elderly were translated into the economic value of the informal care that relatives provided; even if no money was charged, the resource was not considered free.
- Formal care was provided by experts in institutions, whether government-operated or handled by private organizations. The present research regarded formal caregivers as hired caretakers.
- Informal care refers to unpaid caregivers providing constant care and support. It includes care provided by family members, neighbors, and volunteers in the community. The costs of informal care in this study are measured in terms of opportunity costs.

### 2.7. Cost Calculation

The total costs were evaluated by summing the direct and indirect costs that the caretakers paid for one month. The total cost excludes the elderly's earning loss due to their inability to work. It is assumed that the earning loss due to age-related bodily changes has a minimal effect. Cost variable descriptions are given in Table 1.

The direct costs including out-of-pocket expenses for daily supplies, those paid for hired caretakers, and those paid for day/night care in the last month were transformed into yearly expenditure through multiplication by 12.

*Regarding medical devices* (e.g., bathing chairs, wheelchairs, and canes), when an elderly person purchased them, we applied a 5-year useful life to the straight-line depreciation technique to evaluate the annual costs of the medical devices [29, 30].

*The cost of home renovation for elderly* in the previous year was divided by the number of people living in the home to calculate each person's expenditure. The price of home renovation for each person was then divided by 20 years of practical life [29, 30].

With respect to opportunity costs, Suwanrada et al. [12] calculated the salaries on a weekly basis in an effort to determine the value of the time of the respondents who were not working by assigning salaries to them. In this scenario, salary differences could have resulted from age-related and educational level-related differentiation in both genders; consequently, both constraints were used as predictors. Furthermore, Suwanrada et al. [12] considered the coefficients of all the constraints. Moreover, the antilog was multiplied by 52 to calculate the annual income of every subject.

The complete relationship between wage, age, gender, and education level is written as follows:

(1) $$\begin{array}{c} \text{Logit}\left(\text{WAGE}\right)=\beta_{0}+\beta_{1}\cdot \left(\mathrm{AGE}\right)-\beta_{2}\cdot \left({\text{AGE}}^{2}\right)+\beta_{3}\cdot \left(\text{GENDER}\right)+\beta_{4}\cdot \left(\text{EDULEVEL}\right). \end{array}$$

In addition, all estimated costs were further converted into 2017 USD using an exchange rate of USD 1 = THB 34.45 (the exchange rate as of March 31, 2017) [31].

### 2.8. Statistical Analysis

To summarize the features of people who took part in the research, descriptive statistics and sample probability weights were applied.

The entire direct and indirect costs that the aged individual, relative, and caretaker incurred were said to be equal to the price of care of a single individual for 1 month. According to the following theoretical viewpoint and general method, the LTC expenses of a person were considered to entail operational parameters (e.g., gender, education, single or married, lifestyle, disabled or not, income, and any persistent sickness).

Dependent variables were found to be dichotomous as, during the study, the investigators observed a large mass of zero-costs. It presented a skewed distribution, skewed to the right by rare but extremely high-cost events. In addition, applying linear model can result in biased parameters. Subsequently, in this study, the investigators employed a logistic regression instead of the linear model towards the determination of the probability of LTC services [32–34].

Correspondingly, during the study, a binary logistic regression and maximum likelihood function were used for calculation of the log odds ratio: *y* = 0 if no costs were incurred and *y* = 1 if costs were incurred. It was presumed for the analysis that a parametric binary probability model directed the probability of positive expenses Pr (*Y* > 0 *X*).

We selected independent variables potentially associated with LTC costs based on literature reviews. It was presumed that the following factors determine a person's probability of incurring care expenses: age, gender, education, living arrangement, marital status, region, extent of disability, income, and persistent sickness [21, 24, 35–40]. The independent variables were found to be dichotomous.  Table 2 displays the values and corresponding labels.

### 2.9. Ethical Considerations

This research was approved by the Ethics Review Committee for Research Involving Human Research Subjects, Health Sciences Group, Chulalongkorn University, Certificate of Approval number 170/2558. The authors obtained written consent from all participants prior to their involvement in this study.

## 3. Results

### 3.1. Sample Characteristics

Of the rural participants, the mean age was 71.44 years; 47.6% were aged 60–69 years. Of them, 69.1% were accounted for by women; 46% were married but without spouses, while 17.8% were living alone. Around 83.5% alongside half of caregivers had primary education, with 67.3% of them being workless. Nearly 77.8% along with 62.9% of caregivers had an annual income of less than USD 4,354, whereas 90.2% used old age allowance (OAA) for support; 24.9% suffered from a disability, while 81.5% had a chronic disease. About 74.8% had caregivers, with the mean age being 52.82 years; 44.6% were aged 41–60 years. Of the 80% women, 24.5% were unmarried, 85.6% lived with recipients, and 43.9% spent 5–8 hours daily.

Of the urban participants, the mean age was 68.58 years; 59.3% were aged 60–69 years; 65.5% of them were women. Close to 51% were married and had spouses, while 18.8% were single. Also, 63.3% alongside 32.6% of their caregivers had primary education, while 62.3% were workless. Meanwhile, 55.6% had an annual income of less than USD 4,354, while 71.5% depended on OAA; 9.8% had a disability, whilst 75.8% had a chronic disease. In addition, 33.8% of them had caregivers with the mean age of 52.50 years; 48.1% were aged 41–60 years. Nearly 65.2% were women; 57% were married and had spouses, 83% lived with recipients, and 42.2% were workless and without income, whereas 60% spent 5–8 hours daily.

The elderly in the rural area were more likely to have primary education (*χ*^2^ = 44.93, *p* < 0.001) and less likely to be aged 60–69 (*χ*^2^ = 50.32, *p* < 0.001). They were more likely to have an annual income of less than USD 4,345 than those elderly in urban areas (*x*^2^ = 52.84, *p* < 0.001). Moreover, the elderly in the rural areas were more likely to have caregivers (*x*^2^ = 142.50, *p* < 0.001), had OAA as the source of support (*x*^2^ = 47.38, *p* < 0.001), had some type of disability (*x*^2^ = 33.12, *p* < 0.001), and had a chronic disease (*x*^2^ = 4.07, *p* = 0.044) than those elderly in urban areas. (*χ*^2^ = 47.38, *p* < 0.001); they had some type of disability (*χ*^2^ = 33.12, *p* < 0.001), a chronic disease (*χ*^2^ = 4.07, *p*=0.044), and were more likely to have caregivers (*χ*^2^ = 142.50, *p* < 0.001). Caregivers in the rural area were more likely to have primary education (*χ*^2^ = 14.03, *p*=0.001), live with the elderly (*χ*^2^ = 109.14, *p* < 0.001), be workless (*χ*^2^ = 12.54, *p* < 0.001), and spend more time (13–24 hours) daily providing care (*χ*^2^ = 35.57, *p* < 0.001) (Tables 3 and 4).

### 3.2. Annual Cost and Use of Services

The annual cost and use of services are shown in Table 5. The total annual LTC spending was USD 7,285 for rural residents and USD 7,280.6 on average for urban residents.

For the rural area, the average spending on paid caregivers per year was USD 3,309.1 by men and USD 2,612.5 by women; these amounts were less than those for urban men (USD 4,005.8) and women (USD 3,134.9).

The informal care cost of rural residents was USD 2,065.2/year for men and USD 2,145.2/year for women; similarly, the informal care cost of urban residents was USD 2,192.3/year for men and USD 2,021.4/year for women. The cost of day/night care for rural residents was USD 2,717/year for men and USD 1,268.9/year for women.

The cost of daily supplies for rural men was USD 681.2/year and for rural women was USD 664/year. Similarly, the cost of daily supplies for urban men was USD 724.7/year and for urban women was USD 663.3/year.

The average spending on home renovation for the rural elderly was USD 42.7/year and that on medical devices for the rural elderly was USD 24.3/year. The average spending on home renovation for the urban elderly was USD 22.4/year and that on medical devices was USD 18.4/year.

### 3.3. Logistic Regression Analysis to Identify Factors That Influence LTC Utilization

The binary logistic regression analysis results are shown in Table 6. Age was positively associated with the cost of daily supplies (OR = 1.04, 95% CI: 1.01–1.06; *p*=0.010) and that of medical devices (OR = 1.06, 95% CI: 1.03–1.09; *p* < 0.001). However, it was negatively associated with the institutional cost (OR = 0.92, 95% CI: 0.84–0.99; *p*=0.033).

Living in an urban area was negatively associated with the cost of an institutional stay (OR = 0.09; 95% CI: 0.02–0.40; *p*=0.002), the cost of home renovation (OR = 0.69; 95% CI: 0.49–0.94; *p*=0.020), and the informal care cost (OR = 0.48; 95% CI: 0.30–0.77; *p*=0.002).

Being female was negatively associated with the cost of formal care (OR = 0.12; 95% CI: 0.02–0.64; *p*=0.013) and that of home renovation (OR = 1.56; 95% CI: 1.08–2.27; *p*=0.019). Being single was negatively associated with the opportunity cost (OR = 0.59; 95% CI: 0.37–0.94; *p*=0.026). Work was positively associated with the cost of daily supplies (OR = 1.48; 95% CI: 1.00–2.18; *p*=0.048) but negatively associated with the opportunity cost (OR = 0.52; 95% CI: 0.32–0.86; *p*=0.010).

An income of more than USD 4,354 per year was positively associated with the cost of medical devices (OR = 1.77; 95% CI: 1.15–2.73; *p*=0.010) and the cost of home renovation for elderly (OR = 1.53; 95% CI: 1.04–2.26; *p*=0.031).

The presence of at least one of eight chronic diseases was positively associated with the cost of daily supplies (OR = 3.98; 95% CI: 2.18–7.26; *p* < 0.001), the cost of medical devices (OR = 2.24; 95% CI: 1.33–3.80; *p*=0.003), and the cost of home renovation (OR = 1.72; 95% CI: 1.13–2.63; *p*=0.012). Some form of disability was positively associated with the cost of medical devices (OR = 3.44; 95% CI: 2.27–5.22; *p* < 0.001) but not significantly associated with the cost of daily supplies, the day/night care cost, the cost of formal care, the cost of home renovation for elderly, and informal cost.

## 4. Discussion

### 4.1. Annual LTC Costs per Person

This study focuses on the significance of social security and provision of care in costs for the elderly citizens living in the cities and villages of Thailand. Furthermore, this study seeks to explain some very critical determinants of care and its costs.

Noteworthy is the fact that the contribution of the rural areas on average yearly to the total amount of money estimated for this program was USD 7,285. A major share around USD 2,844.7 was distributed to formal care while USD 2,114 was allocated to informal care. The cities' LTC total yearly cost on average was USD 7,280.6. Additionally, the formal and informal sector's yearly economic value of USD 2,380.3 and USD 2,089, respectively, accounted for the bigger share of the total expenditure.

In this study, the hypothesis made is that the total expenditures of the rural elderly are higher than the urban elderly. However, this research does not completely reject the null hypothesis as the total average annual LTC expenditures for both rural and urban elderly are equivalent, according to this research.

This study arrives at a different finding than the several existing types of research, which hypothesized that the expenditure in urban areas is much higher than that of the rural [41, 42]. This observation can be ascribed to the fact that urban areas have better health care and medical facilities compared to the rural areas [14]. In addition, those living in urban areas, owing to their lifestyles, can afford better medical facilities and advanced treatments because they are economically endowed. The low-income people living in rural areas, however, might not be able to even afford low-cost health care [16, 18].

The 2014 Elderly Population survey evidenced 10 million elderly citizens in Thailand, of which 41% were urban residents [43]. In such a scenario, if every citizen were to access all kinds of care such as the ones included in this study, the total care expenditure's approximate value would be around USD 73 billion in average, per year, for the whole country.

The expenditures for both urban and rural contributors, in this study, were compared with six different types of LTC costs. The results showed that the expenditures, on average, on informal and formal care, health care devices, and home makers, were lower in urban areas in comparison to rural areas. However, the day and night care costs, as well as the costs of regular medical supplies in the cities, are higher than that of the rural areas. This could be largely attributed to the different settings in rural and urban areas [16].

When compared to women, the larger part of every category of the rural areas' expenditure is associated with men, save for the informal care. Whereas, in urban areas, men spend higher than women in a day or night care and informal care [21]. Also, according to sociology, this is attributed to a belief in conventional gender roles wherein every gender is culturally assigned roles, for instance, women being assigned families' caregiver roles [7].

Most senior citizens, despite having a choice of established LTC, prefer to remain at home and, thus, obtain support from the formal caregivers or informal programs [44, 45]. Noteworthy is the fact that in developed economies, there are well-sustained formal care expenditures for the senior citizens to access excellent medical and social services. Conversely, the dependent senior citizens of the low- and middle-income economies lack formal care assistance or social security and thereby are dependent on their families [7, 45, 46].

Owing to this situation, many policy initiatives suggest enhancement of abilities of families to provide health security and care to their elderly members. Yet, recent studies from low- and middle-income countries indicate that little attention has been accorded to dependent elderly citizens' informal caregivers as compared to certain informal care for certain diseases [47, 48].

This study concludes that around 70% of caregivers are females, which differed with other studies across Thailand that put it at 57–81% [49, 50]. These results further revealed that majority of the caregivers fall between the age group of 41 and 60 years. Of these statistics, the majority constitutes spouses accounting for 42.2% in urban areas and 40.7% in rural areas. In addition, daughters constitute 31.9% in urban areas and 28.7% in rural areas.

In consideration of sociodemographic variables, majority studies' variations regarding the informal caregivers are indicative of spouses and children to quit their careers towards taking care of the elderly [44, 48].

### 4.2. Factors Influencing LTC Utilization

The results of this study substantially linked the ages of the people to their expenditures on health care devices and regular supplies. The policy-makers' view point suggests that the process of aging could yield unwanted results like (i) higher consumption of health care, (ii) increased dependency, and (iii) higher expenditures on health care. The existing studies indicate that the medical services relating to LTC and acute care are costlier for seniors than it is for the young and the old [35, 51].

Also, an urban center dweller presented an inverse proportional to the use of informal health care, home renovation expenditures, and day and night care. This is evidenced by the fact that people from the rural areas use more of informal care than those from the urban. Additionally, the findings show that there are more home modifications in rural areas than there are in the urban areas. This scenario could be explained by the existence of higher rates of handicap zones (24.9%) in rural areas than in the urban (9.8%). In the past, being married or unmarried was inversely proportional to the use of informal care, which is one factor that affects the financial and mental well-being of people because it impacts on the provision of care and quality of the elderly life [37].

The potential risk of receiving informal care is considerably lower for the elderly who have jobs compared with those who are unemployed. This could be attributed to the fact that people facing health issues might have to quit their careers and need informal care. This means the elderly who are part of the workforce have better health than those who are not working [38].

On average, an elderly person who has an annual income exceeding USD 4,354 spends more on medical care and home renovation for elderly because they have an economical endowment to pay [52]. The study found that OAA assistance is a significant source of income for urban and rural elderly, offering USD 20–30 per month.

This study also found that living conditions and education have minimal effect on the use of health care services. The highly educated are assumed to be more aware of the chronic diseases than the uneducated [20, 40]. Moreover, some form of disability that some people have affects the use of medical devices. For instance, the elderly people having a functional disability often use acute care and formal LTC, leading to an increased health care costs [39, 53].

### 4.3. Strengths and Weaknesses

The major contribution of this study to the current knowledge lies in the provision of unique effects associated with the LTC in cities and villages. This study evaluates the expenditures related to factors which affect the prices of every care. The most desirable consequences of this research are those connected to social resources optimization such as promoting health care volunteer and measures to ensure institutionalization of the older people.

Though this study contributes significantly to the current knowledge in this field, it presents certain limitations. Firstly, this survey is rather cross-sectional and not longitudinal, which demands a cautious use of linkages. Secondly, this study has self-reported information and thereby is subject to bias. Thirdly, some of the important variables were neither optimally coded nor available. For instance, the lack of optimal coded levels of diseases and functional status, which may affect the perfection and thus the modification, may be imperfect. The regression process lacks consideration of the levels of disability. Lastly, the absence of the data on health care volunteer services and occupation details employed an average local formal care wage to derive the approximate value of the informal care. This was done as more than half of the caregivers were female, which might suggest an overestimation of this informal care expenditure.

Despite these limitations, this research will prove important to policy-makers and health workers in their quest to optimize social resources and determine ways of handling this situation and encouraging specific forms of home and community formal care. Moreover, from the social point of view, further studies on longitudinal approaches geared towards assessing the long-term economic effects must be conducted. In addition, further study of LTC policy under the National Health Security funding is desirable.

## 5. Conclusion

This study found the total average annual LTC expenditures between rural-urban residents to be similar. The rapid urban development in coastal areas drives Thailand's economic growth but threatens to leave the rural population behind. In contrast, rural areas surrounding the city are mainly concerned with agriculture that generates lower incomes and these populations are reported to have reduced access to LTC services. The superior ability to pay of urbanites enables them to access better quality health care services, whereas even small LTC expenditures can have a devastating impact on the household economy of rural residents. It is important that to reduce health inequality and to promote greater socioeconomic equality, Thailand must invest in health infrastructure in its rural regions. To meet the needs of increasing populations of disabled elderly people and rising expenditures, countries must restructure their delivery systems and find a balance between the costlier formal and cheaper informal care options.

## Figures and Tables

**Table 1:** Description of cost variables.

Cost type	Cost categories	Description
Direct costs	Daily supplies	Costs associated with medication, special testing, material supplies (feeding tubes, nasal oxygen, urinary catheters, etc.), dressing set, bed pads, adult diapers, tissue paper care, transportation, medical procedure, and physical therapy
	Day/night care	Costs associated with paying for adult day health/day care or overnight care
	Formal care	Costs associated with paying for a licensed practical nurse, a certified nursing assistant, trained caregivers, untrained caregivers, or any kind of paid providers
	Home renovation	Costs associated with various modifications that can make it easier for aging residents to navigate through and live in their homes, including brighter lighting, handrails, stair lifts, and accessible workspaces. These home modifications can range in cost from a few baht for a brighter light bulb to thousands of baht for significant remodeling (stair lifts, etc.)
	Medical devices	Costs associated with back brace, bedpan, blood sugar testing, bubble mattress, chair for bathing, hearing aid, manual home care bed, nebulizers, overbed table, oxygen saturation monitor, oxygen tanks, single cane, suction, tripod cane, walker, and wheelchair

Indirect cost	Informal care (opportunity cost)	The cost of informal care that family members offered without payment. It constituted productivity losses due to lost work time and was estimated using the human capital approach, which measured output losses in lost earnings

**Table 2:** Study variables for logistic regression.

	Value label
*Dependent variables*	
Daily supplies	0 = did not pay, 1 = did pay
Day/night care cost	0 = did not pay, 1 = did pay
Formal care	0 = did not pay, 1 = did pay
Home renovation	0 = did not pay, 1 = did pay
Informal care (opportunity cost)	0 = did not pay, 1 = did pay
Medical devices	0 = did not pay, 1 = did pay

*Independent variables*	
Age	Continuous
Area	0 = rural, 1 = urban
Annual income ≥ USD 4,354	0 = no, 1 = yes
Chronic diseases	0 = no, 1 = present at least one of eight chronic diseases (hypertension, diabetes, stroke, heart, dementia, osteoarthritis, paralysis, or hypercholesterol)
Disability	0 = no, 1 = present at least one of ten disabilities
Education	0 = any education, 1 = no education
Gender	0 = male, 1 = female
Living status	0 = living with other, 1 = living alone
Marital status	0 = married, 1 = single; single includes never married, divorced, widowed, and married but separated
Working status	0 = did not work, 1 = work

**Table 3:** Characteristics of elderly in study area (*N* = 837).

Care recipients	Rural, *N* (%)	Urban, *N* (%)	
*N*	437 (100)	400 (100)	
Mean age (SD)	71.44 ± 7.86	68.58 ± 5.71	
60–69	208 (47.6)	114 (59.3)	*χ* ^2^ = 50.32, *p* < 0.001
70–79	156 (35.7)	154 (38.5)	
≥80	73 (16.7)	9 (2.3)	
Gender			*χ* ^2^ = 1.24, *p*=0.266
Female	302 (69.1)	262 (65.5)	
Marital status			*χ* ^2^ = 6.57, *p*=0.037
Never married	45 (10.3)	47 (11.8)	
Married living together	191 (43.7)	204 (51.0)	
Married not living together^a^	201 (46.0%)	149 (37.3)	
Education level			*χ* ^2^ = 44.93, *p* < 0.001
Primary school and lower degree	365 (83.5)	253 (63.3)	
High school	44 (10.1)	82 (20.5)	
Diploma and higher degree	28 (6.4)	65 (16.3)	
Living arrangement			*χ* ^2^ = 0.11, *p*=0.736
Not alone	359 (82.2)	325 (81.3)	
Working status			*χ* ^2^ = 2.32, *p*=0.128
Did not work	294 (67.3)	249 (62.3)	
Annual income^b^			*χ* ^2^ = 50.79, *p* < 0.001
No income	21 (5.3)	70 (18.7)	
<USD 4,354	311 (77.8)	208 (55.6)	
≥USD 4,354	68 (17.0)	96 (25.7)	
Source of support (not including work)^c^			
Old age allowance	388 (90.2)	284 (71.5)	*χ* ^2^ = 47.38, *p* < 0.001
Children	154 (35.9)	120 (30.2)	*χ* ^2^ = 2.99, *p*=0.084
Pensions/lump sums	30 (7.0)	70 (17.6)	*χ* ^2^ = 22.05, *p* < 0.001
Property income^d^	32 (7.4)	28 (7.1)	*χ* ^2^ = 0.05, *p*=0.829
Other	24 (5.6)	19 (4.8)	*χ* ^2^ = 0.27, *p*=0.607
Disability			*χ* ^2^ = 33.12, *p* < 0.001
Any disabilities	109 (24.9)	39 (9.8)	
Chronic diseases^e^			*χ* ^2^ = 4.07, *p*=0.044
Any chronic disease	356 (81.5)	303 (75.8)	
Caregiver			*χ* ^2^ = 142.50, *p* < 0.001
Have caregiver	327 (74.8)	135 (33.8)	

^a^Married not living together includes separated, widowed, and divorced. ^b^Data were missing for some respondents for the yearly personal income (62). ^c^One person may have more than one source of support. ^d^Property income includes rental income, equity/fixed interest, and return from another investments. ^e^Chronic diseases include hypertension, diabetes, stroke, heart problems, dementia, osteoarthritis, paralysis, and hypercholesterol.

**Table 4:** Characteristics of caregivers (*N* = 462).

	Rural, *N* (%)	Urban, *N* (%)	
*N*	327 (100)	135 (100)	
Mean age (SD)	52.82 ± 15.11	52.50 ± 14.54	
<40	73 (22.3)	31 (23.0)	*χ* ^2^ = 0.78, *p*=0.674
40–59	146 (44.6)	65 (48.1)	
≥60	108 (33.0)	39 (28.9)	
Gender			*χ* ^2^ = 1.49, *p*=0.222
Female	232 (70.9)	88 (65.2)	
Marital status			*χ* ^2^ = 1.30, *p*=0.523
Never married	80 (24.5)	32 (23.7)	
Married living together	198 (24.2)	77 (57.0)	
Married not living together^a^	49 (15.0)	26 (19.3)	
Education level			*χ* ^2^ = 14.03, *p*=0.001
Primary school and lower degree	165 (50.5)	44 (32.6)	
High school	79 (24.2)	37 (27.4)	
Diploma and higher degree	83 (25.4)	54 (40.0)	
Relationship with care recipients			*χ* ^2^ = 3.86, *p*=0.276
Spouse	133 (40.7)	57 (42.2)	
Son/son-in-law	43 (13.1)	21 (15.6)	
Daughter/daughter-in-law	94 (28.7)	43 (31.9)	
Relatives	57 (17.4)	14 (10.4)	
Residence status			*χ* ^2^ = 109.14, *p* < 0.001
Coresidence with elderly	280 (85.6)	112 (83)	
Working status			*χ* ^2^ = 12.54, *p* < 0.001
Did not work	197 (60.2)	57 (42.2)	
Annual income			*χ* ^2^ = 77.21, *p* < 0.001
No income	22 (7.3)	57 (42.2)	
<USD 4,354	190 (62.9)	50 (37.0)	
≥USD 4,354	90 (29.8)	28 (20.7)	
Reason for leaving a job^b^			*χ* ^2^ = 0.90, *p*=0.636
Care for an elderly	96 (55.8)	19 (47.5)	
Retirement	40 (23.3)	11 (27.5)	
Other	36 (20.9)	10 (25.0)	
Time spent in informal caregiving (hours/day)			*χ* ^2^ = 35.57, *p* < 0.001
Less than 4	35 (11.5)	27 (20.8)	
5–8	134 (43.9)	78 (60.0)	
9–12	57 (18.7)	21 (15.6)	
13–24	79 (25.9)	4 (3.1)	

^a^Married not living together includes widowed and divorced. ^b^Data were missing for some respondents for the reason for quitting their job (42).

**Table 5:** Cost on services for rural and urban residents (person/year).

	Rural			Urban		
	Male	Female	Average	Male	Female	Average
Formal care (USD)	3,309.1	2,612.5	2,844.7	4,005.8	3,134.9	2,380.3
*Service use (%)*	37.4	38.7	39.1	42.7	41.2	32.7
Informal care (USD)	2,065.2	2,145.2	2,114	2,192.3	2,021.4	2,089
*Service use (%)*	23.4	31.7	29.0	23.3	26.6	28.7
Day/night care (USD)	2,717	1,268.9	1,590.7	2,438.3	1,741.6	2,090
*Service use (%)*	30.7	18.8	21.8	26.0	22.9	28.7
Daily supplies (USD)	681.2	664	668.7	724.7	663.3	680.6
*Service use (%)*	7.7	9.8	9.2	7.7	8.7	9.4
Home renovation (USD)	34.6	42.3	42.7	21.5	22.6	22.4
*Service use (%)*	0.5	0.6	0.6	0.2	0.3	0.3
Medical devices (USD)	25.0	23.8	24.3	10.5	20.7	18.4
*Service use (%)*	0.3	0.4	0.3	0.1	0.3	0.2
Total cost (USD)	8,841.2	6,756.6	7,285	5,387.4	7,604.7	7,280.6

The exchange rate of USD 1 = THB 34.45 (the exchange rate as of March 31, 2017).

**Table 6:** Factors associated with LTC utilization from logistic regression analysis.

Independent variable	Formal care			Informal care		
	Coefficient	Odds ratio (95% CI)	*p* value	Coefficient	Odds ratio (95% CI)	*p* value
Age (years)	−0.01	0.99 (0.90–1.09)	0.869	0.03	1.03 (1.00–1.06)	0.078
Urban versus rural	−0.63	0.53 (0.12–2.44)	0.416	−0.74	0.48 (0.30–0.77)	0.002
Female versus male	−2.13	0.12 (0.02–0.64)	0.013	−0.17	0.84 (0.52–1.36)	0.479
Single^a^ versus married living together	1.37	3.94 (0.78–19.99)	0.098	−0.53	0.59 (0.37–0.94)	0.026
No education versus any education	1.29	3.64 (0.31–42.78)	0.304	0.45	1.57 (0.67–3.65)	0.296
Living alone	0.21	1.24 (0.22–6.90)	0.809	0.04	1.04 (0.49–2.18)	0.923
Work versus not work	−1.41	0.25 (0.03–2.14)	0.203	−0.65	0.52 (0.32–0.86)	0.010
Annual income ≥ USD 4,354	0.99	2.69 (0.58–12.48)	0.208	0.20	1.22 (0.70–2.12)	0.479
Any chronic disease^b^	−0.72	0.49 (0.11–2.14)	0.342	−0.81	0.44 (0.25–0.80)	0.007
Any disability^c^	0.57	1.77 (0.38–8.38)	0.470	0.04	1.04 (0.62–1.74)	0.895
Constant	−3.20	0.04	0.375	−0.50	0.61	0.663

Independent variable	Day/night care			Daily supplies		
	Coefficient	Odds ratio (95% CI)	*p* value	Coefficient	Odds ratio (95% CI)	*p* value

Age (years)	−0.09	0.92 (0.84–0.99)	0.033	0.04	1.04 (1.01–1.06)	0.010
Urban versus rural	−2.41	0.09 (0.02–0.40)	0.002	0.05	1.05 (0.73–1.51)	0.787
Female versus male	0.09	1.09 (0.35–3.42)	0.884	0.09	1.09 (0.73–1.65)	0.668
Single^a^ versus married living together	0.30	1.35 (0.47–3.85)	0.576	−0.01	0.99 (0.67–1.47)	0.960
No education versus any education	−0.56	0.57 (0.07–4.95)	0.597	−0.15	0.86 (0.42–1.76)	0.683
Living alone	−0.24	0.79 (0.24–2.61)	0.698	0.35	1.42 (0.90–2.24)	0.130
Work versus not work	−0.96	0.38 (0.13–1.15)	0.088	0.39	1.48 (1.00–2.18)	0.048
Annual income ≥ USD 4,354	−0.76	0.47 (0.10–2.15)	0.328	0.30	1.35 (0.87–2.09)	0.184
Any chronic disease^b^	0.89	2.42 (0.52–11.23)	0.258	1.38	3.98 (2.18–7.26)	<0.001
Any disability^c^	−1.84	0.16 (0.02–1.24)	0.080	0.39	1.48 (0.95–2.32)	0.086
Constant	2.71	15.03	0.356	−5.40	0.01	<0.001

Independent variable	Home renovation			Medical devices		
	Coefficient	Odds ratio (95% CI)	*p* value	Coefficient	Odds ratio (95% CI)	*p* value

Age (years)	−0.02	0.98 (0.96–1.00)	0.078	0.06	1.06 (1.03–1.09)	<0.001
Urban versus rural	−0.39	0.68 (0.49–0.94)	0.020	−0.11	0.90 (0.62–1.31)	0.577
Female versus male	0.45	1.56 (1.08–2.27)	0.019	−0.23	0.80 (0.53–1.20)	0.282
Single^a^ versus married living together	−0.03	0.97 (0.68–1.37)	0.857	−0.11	0.90 (0.60–1.35)	0.612
No education versus any education	−0.72	0.49 (0.23–1.04)	0.062	0.09	1.10 (0.54–2.21)	0.797
Living alone	−0.26	0.77 (0.5–1.20)	0.245	−0.29	0.75 (0.44–1.27)	0.281
Work versus not work	0.01	1.01 (0.71–1.43)	0.960	−0.37	0.69 (0.45–1.05)	0.086
Annual income ≥ USD 4,354	0.43	1.53 (1.04–2.26)	0.031	0.57	1.77 (1.15–2.73)	0.010
Any chronic disease^b^	0.54	1.72 (1.13–2.63)	0.012	0.81	2.24 (1.33–3.80)	0.003
Any disability^c^	0.09	1.10 (0.72–1.68)	0.665	1.24	3.44 (2.27–5.22)	<0.001
Constant	−0.05	0.95	0.956	−5.86	0.00	<0.001

^a^Single includes never married, divorced, widowed, and married but not living together. ^b^Any chronic disease includes hypertension, diabetes, stroke, heart problems, dementia, osteoarthritis, paralysis, and hypercholesterol. ^c^Any disability includes personal hygiene, bathing, eating, toileting, upstairs 1-2 step, dressing, walking 200 meters, moving around the bed, urinary incontinence, and fecal incontinence.
